# “Is this injection going to hurt?” Quantifying the pain experience during common lumbosacral spine injections

**DOI:** 10.1016/j.inpm.2025.100632

**Published:** 2025-08-20

**Authors:** Asher Smuek, Rishi Bakshi, Romeo Mays, Lisa Huynh, Joshua Levin, Joshua Rittenberg, Matthew Smuck

**Affiliations:** a Division of PM&R, Department of Orthopaedic Surgery, Stanford University, 450 Broadway St., Redwood City, CA, USA; b Department of PM&R, University of Michigan, Ann Arbor, MI, USA; c Philadelphia College of Osteopathic Medicine, Philadelphia, PA, USA

**Keywords:** Injection, Spine, Patient, Discomfort, Expectations

## Abstract

**Background:**

“Is this injection going to hurt?” Physicians typically answer this from experience since accurate answers are not available in the literature.

**Objective:**

To quantify pain during common lumbosacral spine injections and compare to baseline pain prior to the injections. Analyze differences based on demographic and procedure variables.

**Methods:**

This is a secondary analysis of prospectively collected data from a multicenter trial of patients undergoing bilateral symmetric transforaminal epidural (TFE), facet joint (FJ), or sacroiliac joint (SIJ) injections. Numeric pain ratings (0-10) were obtained at baseline in preop (“What is your current pain?”) and for each injection procedure (“How much did this injection hurt from start to finish?”) first on the right side then the left. Between group comparisons used Chi-squared and ANOVA for categorical and continuous variables, respectively. T-tests compared various pain responses, and multivariate regression determined factors associated with higher procedure pain.

**Results:**

From 244 injections (124 TFE, 60 FJ, 60 SIJ) on 122 consecutive patients (mean age 57.2, 50 % female), age and BMI did not differ between injection groups while sex did (p = 0.001) with more FJ males and SIJ females. Mean baseline pain was statistically equivalent between demographic and injection groups. Mean procedure pain was consistently higher than mean baseline pain, however this difference was small and non-significant for TFE (4.0 vs 3.8) and FJ (3.9 vs 3.3), but larger and significant for SIJ (5.3 vs. 3.6; p = 0.0001). In the multivariate regression analysis only 2 variables remained associated with higher procedure pain, older age (p < 0.0001) and SIJ injection group (p = 0.0021).

**Conclusion:**

The majority of patients (79.1 %) report mild or moderate pain during common lumbosacral spine injections. The average procedure pain of 4.3 on the NPRS scale was only 0.7 points higher than baseline pain recorded in pre-op. Procedure pain from TFE and FJ injections is statistically equivalent to baseline pain and to each other, while SIJ injections produce higher procedure pain with a significant +1.7 point mean increase in pain from baseline. Finally, older adults report significantly greater procedure pain compared to those under 65 years old.

## 1 Introduction

Epidural injections are the most frequently performed interventional pain procedure, followed by injections targeting pain from the facet joints and the sacroiliac joints. Administrative database studies demonstrate consistent growth in the use of these procedures until 2019 when their rates stabilized and then declined [1].

Prior to an injection, the physician must determine that the patient is a good candidate, and the patient must consent to the treatment. During this process, common questions arise. “What are the chances that this is going to help?” The answer to this question is informed by extensive research on the effectiveness of these interventions [[2], [3], [4]]. “What are the risks?” Common side-effects and risks are well documented in the literature. “Is this going to hurt?” Despite their frequency, relatively little is known about the patient experience during these injections. Therefore, physicians typically answer this last question based on anecdotes and experience.

A better understanding of pain experienced during these common interventional procedures can improve patient counseling and assist in setting realistic expectations. Thus, we undertook this study to quantify the amount of pain experienced by patients during the 3 most common lumbosacral pain interventions, and to provide useful context by comparing this to their baseline pain prior to the injections.

## *2* Material and methods

### 2.1 Study design

This study is a secondary analysis of data from a prospective multicenter cohort study. The purpose of the original study was to compare patient reported pain among three different methods of skin anesthesia, finding no differences between them [5]. The current study focuses on the patients’ overall pain experiences during their procedures and compares this to their baseline pain. This study was Institutional Review Boards approved, and Health Insurance Portability and Accountability Act compliant.

### 2.2 Participants

Full details of participant identification and enrollment are provided in the original study [5]. To summarize, patients 18 years or older who were scheduled for bilateral symmetric spine injections were identified from the daily procedure schedule and approached for enrollment after arriving in pre-op. Enrollment required bilateral symmetric procedures based on the primary aim of the original study, to compare different methods of skin anesthesia. No significant differences were found [5]. A total of 127 consecutive consenting patients were enrolled in the original trial. From this cohort, the current study excluded patients who received injections outside the lumbar or sacral spine, and those who received injections other than transforaminal epidural (TFE), facet joint (FJ), or sacroiliac joint (SIJ) injections. FJ injections in the original study included symmetric medial branch nerve blocks and intra-articular injections.

### 2.3 Study outcomes

Participant demographic data (age, sex and BMI) were collected along with the type and location of each injection, treatment needle gauge, and use of intravenous sedation (yes or no). Participant pain was collected using an 11-point numerical pain rating scale (NPRS) of 0–10, with 0 being “no pain” and 10 being “the worst pain of your life.” Following enrollment and consent in pre-op, baseline pain was determined immediately after consent in the pre-op room by instructing participants to rate their current pain using the NPRS and asking, “What is your current pain?” Next, they were informed that the same NPRS would be used to measure pain experienced during the injection procedure. In the procedure room, for each participant the injection started on the right side. After this injection was completed and immediately after the treatment needle was removed, the participant was asked to rate the procedure pain using the same NPRS and the question, “How much did this injection hurt from start to finish?” Next, the procedure was repeated on the left side following the same steps described above to document the procedure pain score for the left side. Thus, with all participants receiving bilateral symmetric procedures, each provided one baseline NPRS and two procedure NPRS ratings (one right and one left).

### 2.4 Data analysis

Participant demographics and procedure details were summarized using descriptive statistics. For categorical variables, the count (n), denominator (N) and percentages were calculated. For continuous variables, the mean and standard deviation (SD) were determined. Injection procedures were separated into 3 groups by injection type (TFE, FJ, and SIJ). In addition, comparisons were made based on demographics, sedation use, and between the first injection (right) and second injection (left). Evaluation for statistical differences between groups used Chi-squared and ANOVA for categorical and continuous variables, respectively. Within group comparisons of baseline pain to procedure pain used a paired T-test while comparisons of pain between groups used an un-paired T-test. Multivariate regression analysis determined relationships between multiple variables. Age and BMI were treated as a continuous measure in the multivariate analysis, otherwise they were categorized. Age was divided into 3 categories: younger (18 to <45), middle (45 to <65) and older (65+). BMI used 2 categories: obese (BMI ≥30) and not (BMI<30). In addition to evaluating pain as a continuous variable, additional analyses were performed categorizing pain severity as mild (<4), moderate (4–7), or severe (>7) [6,7], Finally, changes in pain from baseline to procedure pain were evaluated relative to a 2-point minimum clinically important change (MCIC) in the NPRS [8,9]. All statistical testing employed a 0.05 significance level.

## 3 Results

### 3.1 Cohort demographics and injections

**Fig. 1:**
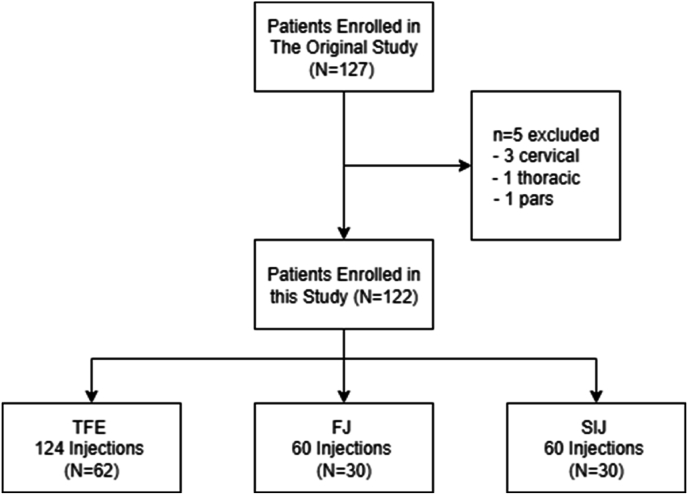
Diagram of patient flow in this study, and composition of the 3 injection groups: transforaminal epidural (TF), facet joint (FJ), and sacroiliac joint (SIJ) injections.

From the 127 consecutively recruited participants in the original study, 122 were retained for this analysis with 5 eliminated based on the exclusion criteria, as shown in Fig. 1. Participant baseline demographic and clinical details are provided in Table 1. Since all recruited patients received symmetric bilateral single-level procedures this resulted in a total of 244 injections in this analysis, including 122 TFE, 60 FJ, and 60 SIJ injections. Using a 3-way Anova there were no differences between the 3 injection groups in participants’ age (p = 0.1698) or BMI (p = 0.7798). Conversely, sex was significantly different (p = 0.0015) based on a Chi-square test with the TFE group having equal parts males and females (M = 31, F = 31), the FJ group having more males (M = 22, F = 8), and the SIJ group having more females (M = 8, F = 22).

**Table 1:** Demographics.

	N = 122
Age (mean, SD)	57.254, (16.8)
Female (n, %)	61 (50)
BMI (mean, SD)	29.29 (7.22)
White (n, %)	109 (89.3)
Black	5 (4.1)
Asian/Pacific Islander	4 (3.3)
Asian Indian	1 (0.8)
Other	3 (2.5)

### 3.2 Baseline and procedure pain

Baseline pain, (“What is your current pain?” asked in pre-op before entering the procedure room) for the entire cohort was 3.6 on average, with no significant differences in average baseline pain between the 3 injection groups (TFE = 3.8, FJ = 3.3, and SIJ = 3.6; 3-way Anova p = 0.3818). Furthermore, baseline pain did not differ based on sex (95 % CI -0.513 to 0.775, p = 0.6888) or BMI (95 % CI -1.222 to 0.119, p = 0.1065). However, age category did have a significant impact with highest mean baseline pain of 5.7 in those age 18 to <45, followed by 3.4 in those age 45 to <65, and 2.7 in those 65+ (p < 0.00001).

Average procedure pain (“How much did this injection hurt from start to finish?”) was 4.3 for the entire cohort, 4.1 for TFE, 3.9 for FJ, and 5.3 for SIJ injections. Binary comparisons between the injection groups revealed significantly higher pain in the SIJ group when compared to TFE (95 % CI -2.030 to −0.531, p = 0.0009) and FJ (95 % CI -2.43 to −0.51, p = 0.0031) and no difference in pain between TFE and FJ groups (95 % CI -0.527 TO 0.900, p = 0.6071). Again, age category had a significant impact with highest mean procedure pain of 5.7 in those 65+, followed by 4.1 in those age 45 to <65, and 2.5 in those age 18 to <45 (p < 0.00001).

The association between needle gauge and procedure pain was not analyzed separately since 25-gauge or 26-gauge needles were used for all TFE and FJ injections while 22-gauge needles were used exclusively during SIJ injections. Mean procedure pain did not differ between obese and non-obese BMI groups (95 % CI -0.610 to 0.709, p = 0.8826), but was significantly different between females and males, 4.8 vs 3.9 respectively (95 % CI -1.526 to −0.286, p = 0.0044). However, this finding did not control for the significant differences in sex proportions between the 3 injection groups where there were fewer males in the SIJ group (associated with the highest average pain) and more males in the FJ group (associated with lowest procedure pain). Controlling for this in the multivariate regression analysis only 2 variables remained associated with higher procedure pain, age (p < 0.0001) and SIJ injection group (p = 0.0021), with no significance for BMI or sex.

### 3.3 Change in pain from baseline to procedure

**Fig. 2:**
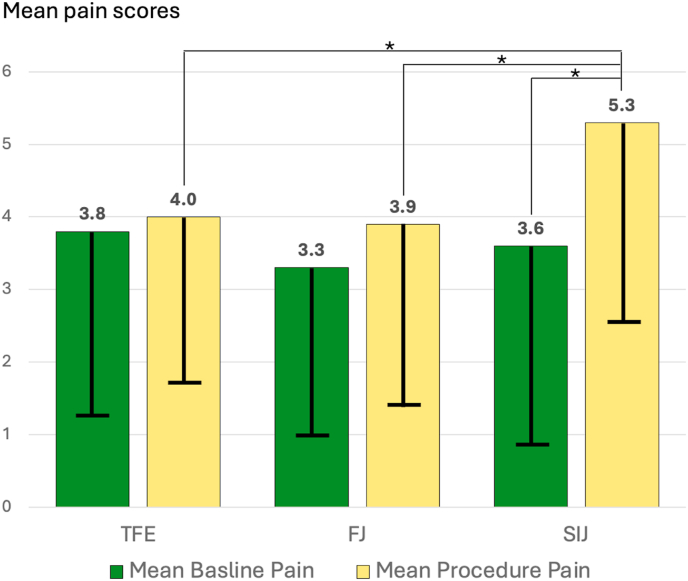
Bar graph showing mean baseline pain (green bars) and mean procedure pain (yellow bars) for each of the 3 injection groups: transforaminal epidural (TF), facet joint (FJ), and sacroiliac joint (SIJ) injections. Error bars display the standard deviation within each bar and the numerical mean value is provided above. Wisker lines with asterisk (∗) denote statistically significant differences (p < 0.05) between the connected bars. Baseline pain was not significantly different between the 3 injection groups. Procedure pain was significantly higher for SIJ compared to TFE and FJ. Procedure pain was significantly higher than baseline pain for SIJ only.

From the findings above, average change in pain from baseline to procedure was calculated. The change of +0.7 points for the entire cohort was statistically significant (95 % CI -1.051 to −0.298, p = 0.0005), however, this result was not consistent between the 3 injection groups. Specifically, the average change in pain from baseline to procedure was small and non-significant for the TFE (+0.2; 95 % CI -0.730 to 0.302, p = 0.4141) and FJ (+0.5; 95 % CI -1.40 to 0.23, p = 0.1564) while it was larger and significant for the SIJ group (+1.7; 95 % CI -2.43 to −1.00, p < 0.0001) as shown in Fig. 2.

Applying the 2-point MCIC to the change from baseline pain to procedure pain, 36.9 % (n = 90) of injections overall met or exceeded the MCIC. The proportion meeting or exceeding the MCIC in each of the 3 injection groups was: TFE = 30.3 % (n = 37), FJ = 36.7 % (n = 22), and SIJ = 51.7 % (n = 31). The between group differences were significant comparing TFE to SIJ (p = 0.0040) and not significant comparing TFE to FJ (p = 0.3522) and FJ to SIJ (p = 0.0980).

### 3.4 Procedure pain severity

**Fig. 3:**
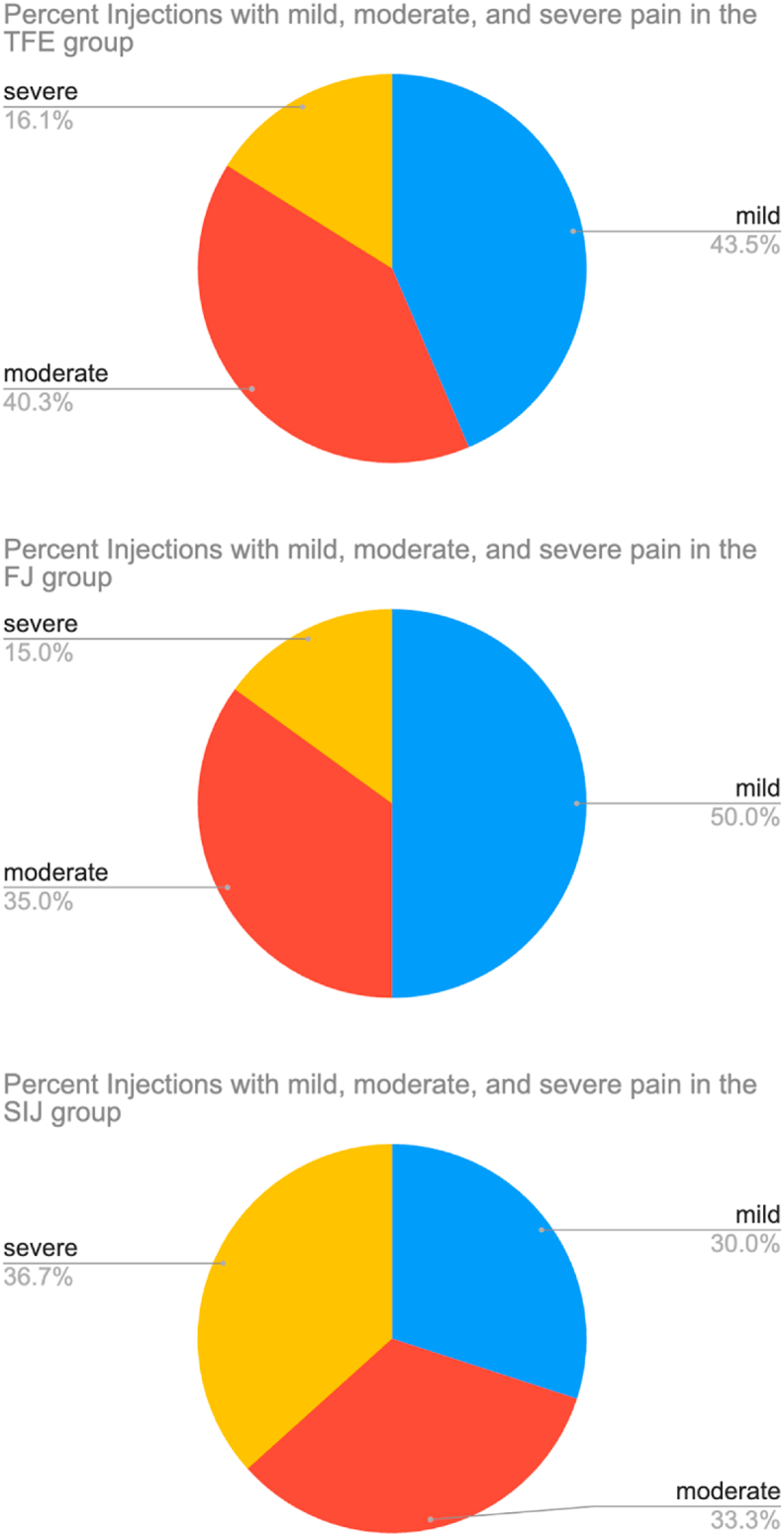
Pie graph demonstrating proportions of injections with mild (<4), moderate (4–7) and severe (>7) pain for each of the 3 injection groups: transforaminal epidural (TF), facet joint (FJ), and sacroiliac joint (SIJ) injections. Chi-square reveals statistically significant differences (p = 0.0025) between the groups.

The number of injections with mild (<4), moderate (4–7) or severe (>7) procedure pain was also considered. Overall, mild procedure pain was most common (41.8 %; n = 102), followed by moderate pain (37.3 %; n = 91), and severe pain (20.9 %; n = 51). Among the 3 injection groups, using chi-square to compare the differences in the proportions with mild, moderate and severe pain there was a statistically significant difference (p = 0.0025). Further details are provided in Table 2 and Fig. 3.

**Table 2:** Proportion of injections reporting mild, moderate, and severe pain according to group and overall (%).

Group	TFE (%)	FJ (%)	SIJ (%)	Overall (%)
Mild	43.5	50	30	41.8
Moderate	40.3	35	33.3	37.3
Severe	16.2	15	36.7	20.9

### 3.5 Sedation

Eighteen patients (36 injections) elect to receive sedation during their procedures. There were no differences in baseline pain between those who received sedation and those who did not, both overall and within the 3 injection groups. Likewise, there were no significant differences in procedure pain between injections completed with sedation and without sedation for all 3 injection groups: TFE (95 % CI -0.963 to 1.494, p = 0.6696), FJ (95 % CI -1.98 to 1.18, p = 0.6150), and SIJ (95 % CI -0.25 to 4.55, p = 0.0782).

### 3.6 First versus second side injections

All patients received symmetric bilateral single-level injections, starting on the right side and finishing on the left. Comparing these, overall, the mean procedure pain was slightly higher on the second side by 0.2 points (right = 4.2 vs left = 4.4), however this difference was not statistically significant (95 % CI -0.479 to 0.782, p = 0.6360). Likewise, there were no differences in procedure pain between the first and second side injections for any of the 3 injection groups: TFE 4.1 vs 4.0 (95 % CI -0.874 to 0.696, p = 0.8234), FJ 3.6 vs 4.1 (95 % CI -0.44 to 1.31, p = 0.3197), and SIJ 5.1 vs 5.5 (95 % CI -0.37 to 1.10, p = 0.3173).

## 4 Discussion

For physicians who perform spine injections, this study provides quantitative and contextualized information to help answer the common question, “How much will this injection hurt?” For the 3 most common lumbar spine interventions, we found the average procedure pain was 4.3 on the 11-point NPRS. This was less than 1-point higher than average baseline pain reported before the procedure. In other words, patients can be advised that the pain experienced during these procedures is, on average, less than 1-point greater than the pain they have currently while waiting for the injection in pre-op. However, approximately 1 in 3 injections (36.7 %) produced an increase of pain from baseline that exceeded the 2-point MCIC, and approximately 1 in 5 injections (20.9 %) caused severe pain (pain >7). For SIJ injection the proportion is higher with slightly more than 3 in 10 injections (36.7 %) causing severe pain. Also, older adults report higher procedure pain.

Prior studies on procedural pain during lumbar spine injections focused on the impact of variable methods employed by clinicians to mitigate pain. As expected, smaller gauge needles caused less pain than larger needles [5,10]; and single needle techniques were less painful than those using multiple needles [11]. While the use of topical or subcutaneous local anesthetic can improve pain during the initial phase of an injection, it does not reduce overall procedure pain [5,12,13]. Likewise, alternatives to subcutaneous local anesthesia such as mechanodesensitization were less painful during the initial phase of an injection but do not improve overall procedure pain [14]. Deep administration of local anesthetic was no better than superficial alone [15], and aromatherapy had no impact on procedure pain [16].

We also explored the impact of patient and procedural variables. For example, while a greater length of needle is needed to reach the intended target in obese patients, procedure pain was no different between the obese and non-obese groups. We suspect that this is because, in our experience, patients seldom report pain as needles advance through adipose tissue. Sex did not relate to procedure pain severity in the multivariate analysis. All patients received bilateral, symmetric, single-level injections allowing a comparison of pain between the first and second sides, finding no significant difference. Asking patients about pain on one side and then the other could result in anchoring, decreasing the reliability of procedural pain reporting on the second side. Patients could elect to receive minimal sedation during their injections, and 15 % chose to do so. There were no differences in procedure pain between patients who requested and received sedation and those who did not. While the specific details of sedation medications were not recorded, both practices that enrolled patients into this study exclusively used versed with or without fentanyl, and they rarely exceed doses greater than 2 mg of the former and 50mcg of the latter with all patients remaining alert throughout their procedures.

Age was a factor, with the oldest group (65+) having both significantly lower baseline pain and higher procedure pain. Many conditions that cause chronic pain are more prevalent in older adults, yet pain sensitivity is reduced in older age [17]. This occurs alongside age-related reductions in the structure and function of brain regions involved in pain processing [18]. This may explain why the older participants reported lower baseline pain. Furthermore, differences in baseline pain may be due to divergent pathologies in the young and the old. For example, radicular pain in younger subjects is commonly caused by lower lumbar disc herniation, whereas in the elderly it is more often due to lumbar stenosis. Symptoms from stenosis classically aggravate with standing and improve with sitting, while the opposite is true of lumbar disc herniation, and all patients were seated or recumbent during the baseline pain assessment. Higher procedure pain in the older group runs counter to their lowered pain processing and higher pain thresholds. We speculate this finding is due to progressive lumbar degeneration creating more anatomic obstacles and challenges, lengthening procedures and increasing the number of passages and attempts often needed to complete injections in the elderly.

Patients reported significantly higher procedure pain with SIJ injections than with TFE or FJ injections, respectively 5.3 versus 4.1 versus 3.9. We can think of a few potential reasons for higher pain during SIJ injections. First, larger 22-gauge needles were used for SIJ injections while smaller 25-gauge or 26-gauge needles were used for all TFE and FJ injections. Second, compared to TFE and FJ, SIJ access involves passage through the dense posterior sacroiliac ligaments and substantially more bone contact during needle navigation into the articular portion of the joint. In addition, injection inside a diarthrodial joint can cause pain during insufflation. The larger proportion of female patients in the SIJ group is not a factor since sex was not related to pain severity in the multivariate analysis, and studies outside the spine similarly found that injection procedure pain did not differ between females and males [19,20].

There are several limitations to this study. First, this study is a secondary analysis of data from a prospective study and is limited to the variables collected in the primary dataset. Therefore, we cannot provide information on several variables of potential interest including patient expectations regarding procedure pain, dosage of sedation when used, routine pain medications used, psychological comorbidities, procedure time, number of attempts, and impact of multiple level versus single level injections. The generalizability of these results is unknown given the numerous variables involved in an injection procedure that were not captured in this dataset, such as proceduralist experience, presence of trainees, alternate techniques, injectate speed, type and concentration of local anesthetic, catastrophizing, rapport and others. For example, in this study all patients were recruited from two academic spine centers so understanding the generalizability of these results requires additional research in different geographic and clinical settings. That said, similar average procedure pain, ranging from 3 to 5 points on the NPRS, was reported in several prior studies focused on methods to mitigate procedure pain [12,15,16]. suggesting broader generalizability of our study findings. Lastly, patients in this study reported an average baseline pain level of 3.6 pre-procedure. We speculate that this is lower than their average daily pain or daily worst pain, but neither was provided in the dataset. We think that comparisons of procedure pain to average daily pain or worst daily pain, instead of current pain in pre-op, would yield different results.

## 5 Conclusions

The majority of patients (79.1 %) report mild or moderate pain during common lumbosacral spine injections. The average procedure pain of 4.3 on the NPRS scale was only 0.7 points higher than current baseline pain recorded in pre-op. Procedure pain from TFE and FJ injections is statistically equivalent to baseline pain and to each other, while SIJ injections create higher procedure pain with a significant 1.7 point mean increase in pain from baseline. Finally, older adults report significantly greater procedure pain compared to those under 65 years old.

## Declaration of competing interest

The authors declare the following financial interests/personal relationships which may be considered as potential competing interests: Matthew Smuck reports a relationship with BlueJay Mobile Health that includes: consulting or advisory. Matthew Smuck reports a relationship with Relievant Medsystems Inc that includes: funding grants. Matthew Smuck reports a relationship with Axial Consulting Group that includes: equity or stocks. Josh Levin reports a relationship with Scilex Pharmaceuticals that includes: consulting or advisory. Matthew Smuck serves as an Associate Editor for the journal. Given his role as an Associate Editor had he had no involvement in the peer review of this article and had no access to information regarding its peer review. Full responsibility for the editorial process for this article was delegated to another journal editor. If there are other authors, they declare that they have no known competing financial interests or personal relationships that could have appeared to influence the work reported in this paper.
